# Kinetics and Thermodynamics of Pb(II), Zn(II), and Cd(II) Adsorption from Aqueous Solutions onto Activated Biochar Obtained from Tobacco Waste

**DOI:** 10.3390/ma18102324

**Published:** 2025-05-16

**Authors:** Beata Jabłońska, Paweł Jabłoński, Jerzy Gęga

**Affiliations:** 1 Department of Environmental Engineering and Biotechnology, Faculty of Infrastructure and Environment, Czestochowa University of Technology, Brzeźnicka St. 60a, 42-200 Częstochowa, Poland; 2 Department of Automation, Electrical Engineering and Optoelectronics, Faculty of Electrical Engineering, Czestochowa University of Technology, Armii Krajowej 17, 42-200 Częstochowa, Poland; pawel.jablonski@pcz.pl; 3 Department of Materials Science and Engineering, Faculty of Production Engineering and Materials Technology, Czestochowa University of Technology, Armii Krajowej 19, 42-200 Częstochowa, Poland; jerzy.gega@pcz.pl

**Keywords:** adsorption, activated carbon, tobacco stems, tobacco waste, lead, zinc, cadmium

## Abstract

Waste tobacco stems from the tobacco industry were used to obtain activated carbon by thermal conversion and chemical activation with KOH. The aim was to investigate its adsorption ability towards Zn(II), Cd(II), and Pb(II) from aqueous solutions. Fundamental physical and chemical properties were investigated, and the point of zero charge pH was detected. The results showed that the obtained activated carbon was characterized by a high specific surface area, pore volume, and negative surface charge, which could make it an efficient metal adsorbent. In the next step, the optimal adsorption conditions were determined using Central Composite Design. Finally, the adsorption kinetics and thermodynamics were studied. The adsorption rate is very high for Pb(II) and Cd(II), whereas it is noticeably lower for Zn(II). The negative value of Gibbs free energy change (∆G) confirmed that the adsorption process of the tested metal ions is feasible and proceeds spontaneously. The thermodynamics indicate that the adsorption of zinc and lead on the tested carbon is an exothermic process, and for cadmium, this process is endothermic.

## 1. Introduction

Water deficit, as well as poor water quality, has resulted in a global water crisis [1]. Without changes in water management, the water crisis will deepen, exacerbating existing inequalities. According to a report by the European Investment Bank, around 380 billion m^3^ of wastewater is generated worldwide annually, which is an alarming increase compared to previous years. Forecasts indicate that by 2030 this number will increase to 470 billion m^3^, and by 2050, it will reach 574 billion m^3^ [2]. The increase in the production of wastewater, which pollutes water and soil, has mainly been caused by industrialization and urbanization. Industrial and municipal wastewater is a common source of contamination of surface and groundwater with various harmful compounds, including heavy metals. Metal mining, smelting, foundries, and other metal-based industries also create large amounts of waste and wastewater containing trace elements [3]. Ore mining processes such as crushing, flotation, and roasting generate chemicals that can pollute the environment, posing a serious threat to aquatic ecosystems and human health, especially since metals are not biodegradable. In particular, heavy metals such as lead, zinc, or cadmium can accumulate in aquatic organisms and enter the food chain [4]. As a result, many methods for removing metal ions from water and wastewater have been developed, including chemical precipitation [5], coagulation and flocculation [6], ion exchange [7], membrane separation processes [8], adsorption [9], and others.

The sustainable management of water resources in the world requires ensuring access to clean drinking water and implementing innovative water treatment technologies. Adsorption is one of the most often used technologies, and the most common adsorbents include activated carbons obtained from hard coal, peat, or brown coal, as well as carbon nanotubes, graphene, and natural clays [10]. In recent years, carbon materials obtained from plant biomass (biochars) have gained significant importance in the adsorption method for removing pollutants from water [11]. Due to their ecological properties and low price, they have become a promising adsorbent compared to expensive activated carbons produced from synthetic fuel precursors. The production of commercial activated carbons is associated with high costs, which limits their use in fields such as environmental engineering, agriculture (as fertilizers), or construction engineering (as composites). In turn, biochars can be produced at minimal cost from easily available agricultural waste [12]. Such a solution enables the recycling of resources, which not only reduces the amount of waste but also contributes to saving natural resources and reducing carbon dioxide emissions. Activated biochars are obtained in thermochemical conversion processes, most often as a result of the high-temperature pyrolysis of biomass without access to oxygen, as well as hydrothermal carbonization, gasification, or torrefaction. In the pyrolysis process, organic matter is decomposed, volatile components are removed, and a porous structure is created. Further activation of the material (e.g., with steam, oxidizing gases, or chemical compounds) increases the number and size of pores and, thus, the surface available for the adsorbate. Depending on the type of original biomaterial, the conditions of synthesis, and the methods of physical and chemical activation, activated carbons differ significantly in terms of physicochemical properties (e.g., specific surface area, porosity, elemental composition, presence of functional groups, pH value of the zero-charge point, etc.) [13]. It is crucial to transform existing knowledge into effective and practical water treatment systems, including the use of biochar technologies that promote savings and the protection of natural resources. Currently, research on the use of biochars focuses on developing methods for their production, studying their properties, and determining the applications of the obtained biochar products in the context of thermochemical processes. This approach is in line with the “waste-to-treasure” concept and supports activities aimed at CO_2_ neutrality, resource recycling, energy saving, and emission reduction [14].

A potential source of biochar is tobacco (*Nicotiana tabacum* L.)—a plant widely cultivated worldwide, mainly for the production of tobacco products such as cigarettes, cigars, and snuff. According to [15], global tobacco production was 6.4 million tons in 2023. The largest tobacco producer is China, which produces about 2.4 million tons of tobacco per year, followed by India, Brazil, and Zimbabwe. It is estimated that more than 200 million tons of waste is generated annually in the process of tobacco cultivation and cigarette production [16]. This waste includes by-products generated during the cultivation, production, and processing of tobacco products, such as stems, dust, and tobacco leaf residues that can still be smoked. Tobacco stems, unlike crop straw (wheat or rye stalks), are difficult to biodegrade in the natural environment. For this reason, they are usually burned in the open after the leaves have been harvested. This action leads to the pollution of the atmosphere and groundwater as a result of the release of harmful nicotine [17,18]. Therefore, it is important to develop effective methods of managing this waste, which will allow for its proper treatment and transformation into resources. Biomass from tobacco waste can be used to produce valuable bioproducts, such as biofuels [19,20], carbon materials [21], or natural fertilizers [22,23]. The transformation of tobacco waste into bioproducts creates new opportunities in the tobacco industry, improving its sustainability while also benefiting society by contributing to environmental protection and the development of an economy based on renewable resources [24,25].

The process of extracting bioactive compounds from tobacco waste [26,27], the production of fermentable sugars from tobacco stems [28], and the use of biochar from tobacco stems as a fertilizer to improve the physical properties of soil [22,29] have been described in detail in the literature. However, little is known about the use of activated carbons obtained by the thermochemical conversion of these wastes in drinking water treatment processes, especially as adsorbents of pollutants. Currently, the use of biochar obtained from plant biomass in water treatment systems is limited. This is due to the fact that biochars are heterogeneous materials obtained from different types of biomass. Differences in their properties, such as pH, ionic strength, organic matter content, and other factors, make the pollutant sorption process more complex than in the case of commercial activated carbon [30]. Therefore, each biochar requires individual research to determine its properties and capabilities for the sorption of various pollutants. The aim of this work was to investigate the sorption properties of activated carbon obtained by the thermochemical transformation of waste tobacco stems in relation to three heavy metals: Zn(II), Cd(II), and Pb(II). The optimal conditions for the sorption of these compounds were determined, and studies on the sorption kinetics and thermodynamics were carried out, which will allow for the practical application of such activated carbon in water treatment systems.

## 2. Materials and Methods

### 2.1. Materials

**Figure 1:**
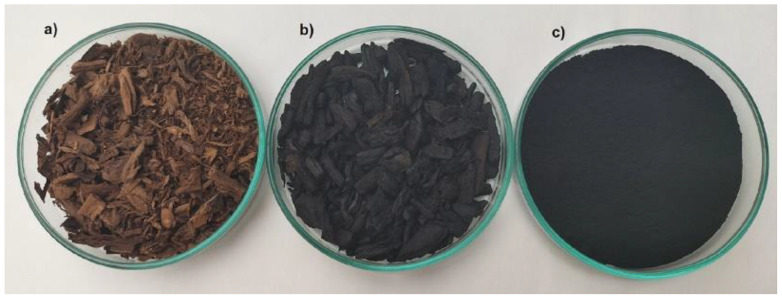
Image of the biomass used: (**a**) tobacco waste stems; (**b**) biochar obtained by slow pyrolysis at 400 °C; and (**c**) activated carbon obtained after chemical modification (TWAC).

Biomass from waste tobacco stems obtained from the tobacco industry in Poland was used (Figure 1a). Activated carbon from tobacco waste was prepared according to the literature [31], with minor modifications, as described below. The biomass was subjected to slow pyrolysis at a temperature of 400 °C with a heating rate of 5 °C/min and a reaction time of 120 min. Pyrolysis was carried out in a horizontal tube furnace (PRW-S100 × 780/11 by Czylok Sp. z o.o., Jastrzębie Zdrój, Poland) with a nitrogen stream (5 L/min). The obtained biochar (Figure 1b) was chemically activated using KOH. For this purpose, 1 g of biochar was mixed with 2 g of KOH, and 100 mL of demineralized water was added, and then, the sample was stirred for 24 h (400 rpm). After this time, the biochar sample was dried at 105 °C, placed in a nickel boat, and subjected to pyrolysis in a nitrogen stream (5 L/min). The temperature in the furnace was increased at a rate of 5 °C/min to the activation temperature of 800 °C, which was maintained in the furnace for 2 h. After the pyrolysis process was completed, the material was left in the reactor until room temperature was reached. Then, it was flooded with 5% HCl and left for 24 h. The sample was washed abundantly with hot distilled water to pH ≈ 7 in the effluent and dried at 105 °C to a constant mass. The activated carbon prepared in this way (Figure 1c) was stored in a glass container and marked as TWAC (tobacco waste activated carbon).

Three metals were selected for sorption studies, i.e., Pb(II), Zn(II), and Cd(II), which are most frequently found locally in surface and groundwater, raw and treated sewage, and even in drinking water [32,33,34,35] as a result of their infiltration with contaminated sewage. Solutions of the heavy metals Zn(II), Cd(II), and Pb(II) were prepared from zinc nitrate (Zn(NO_3_)_2_·6H_2_O), cadmium nitrate (Cd(NO_3_)_2_·4H_2_O), and lead nitrate (Pb(NO_3_)_2_ analytical grade), which were purchased from Pol-Aura Sp. z o.o. (Zawroty, Poland).

### 2.2. Methods

#### 2.2.1. Measurements

The concentration of metal ions in water extracts and samples after sorption was analyzed using an inductively coupled plasma–atomic emission spectrometer type ICP-AES. The determination of pH was carried out using the pH meter pH/mv CP-401 (ELMETRON, Zabrze, Poland) with an accuracy of ±0.002 pH. The content of basic elemental components (C, H, N, S) was determined on an elemental analyzer type LECO Tru/Spec CHN/S. The specific surface area, pore volume, and size distribution were determined based on the course of the nitrogen vapor adsorption/desorption isotherm at 77 K on an ASAP 2020 micrometer analyzer (Micrometrics, Atlanta, GA, USA). The distribution of functional groups in the tested adsorbent was assessed using a Fourier-transform infrared spectroscopy (FT/IR-6200, Jasco, Heckmondwike, UK). FTIR spectra were recorded in the range from 4000 to 400 cm^−1^. Infrared spectra were collected using an ATR attachment.

#### 2.2.2. Physicochemical Analyses of Activated Carbon

The tested activated carbon was characterized in terms of physicochemical properties such as pH, moisture, ash, volatile substances, bulk density, iodine value, and methylene blue index. The water pH was measured in aqueous solutions by the potentiometric method using a pH meter. The total moisture content ($H_{a}$) was assessed in accordance with the standard [36]. The determination consisted of determining the weight loss of the tested material as a result of its drying at 105 °C. The determination of ash content ($A_{a}$) in the tested samples was performed in accordance with the standard [37]. The determination consisted of burning 1 ± 0.1 g of TWAC in a furnace at a temperature of 815 ± 10 °C for 90 min in an FCF22S muffle furnace (CZYLOK, Jastrzębie_Zdrój, Poland) and measuring the mass loss after incineration of the sample. The ash content in the sample was expressed in terms of the air-dry state. The bulk density was determined in accordance with the standard [38].

The volatile matter (VM) content was determined in accordance with the standard [39]. The determination consisted of placing samples with TWAC in an oven heated to 850 ± 10 °C for 7 min, cooling the sample, and measuring the mass. The volatile matter content was converted to a dry and ash-free state as follows:

(1) $$VM\left[\%\right]=\left(\frac{∆m}{m}\cdot 100-H_{a}\right)\cdot \frac{100}{100-\left(H_{a}+A_{a}\right)},$$

where Δm is the mass loss between the weighed portion and the final sample, g; m is the mass of the TWAC sample used for measurement, g; $H_{a}$ is the moisture content in the analytical sample, %; and $A_{a}$ is the ash content determined in the analytical sample, %.

The efficiency of the biochar modification process was assessed based on the available surface area and estimated by performing a standardized determination of the iodine adsorption number (iodine value) in accordance with [40]. The methyl number was determined according to [41].

The H/C ratio defining the hydrophobic nature of the solid and the O/C ratio defining the presence of functional oxygen groups were calculated from the elemental composition of the tested activated carbon. The oxygen content was determined by the calculation method based on the difference according to the following equation:

(2) $$O\left(\%\right)=100\%-\left[A_{a}\left(\%\right)+C\left(\%\right)+N\left(\%\right)+H\left(\%\right)+S\left(\%\right)\right].$$

#### 2.2.3. Point of Zero Charge pH

The pH_pzc_ is the pH value at which the surface of a solution or suspension of a solid in water has an electric charge of zero. The pH_pzc_ of the adsorbent material was determined in accordance with [42] using the constant addition method. Eleven 100 mL glass bottles were prepared with 50 mL of 0.01 M NaCl solution in each. By adding 0.1 M HCl or 0.1 M NaOH solution, the pH values of the tested solutions were set in the range of 2 to 12. The bottles were tightly closed and left for 2 h to stabilize the pH of the solution, after which the pH of the solution was measured (initial pH). Then, 0.15 g of the tested carbon was added to the solutions with the appropriate initial pH and flushed with nitrogen gas to remove CO_2_ from the solution, and then, the bottles were tightly closed. After that, the bottles with the solutions were shaken for 24 h at 200 rpm using an orbital shaker type RS-OS 5 and then set aside. After 72 h of equilibration at room temperature, the final pH of the solution was measured. The pH_pzc_ was determined by plotting a curve of the difference between the final pH and initial pH against the initial pH.

#### 2.2.4. Sorption Studies

Adsorption studies of Zn(II), Cd(II), and Pb(II) were carried out in single-component aqueous solutions consisting of deionized water and the tested compounds. In the first step, the optimal adsorption conditions were established. The initial concentrations of Zn(II), Cd(II), and Pb(II) were assumed to be in the range of 10 to 90 mg/dm^3^. The TWAC doses were assumed to be in the range of 1 to 6 g/L, and the pH was assumed to be in the range of 3−6. Each metal of the appropriate concentration and a volume of 100 mL of distilled water were introduced into conical flasks. Samples prepared in this way were shaken on a rotary shaker for an hour at 20 °C, after which the vessels were placed in a dark room for 23 h. After this time, the water from the conical flasks was decanted and centrifuged in a centrifuge (MWP-2 type) at 2500 rpm. The efficiency of the Zn(II), Cd(II), and Pb(II) adsorption process was evaluated as the percentage removal (PR) of the tested contaminants as follows:

(3) $$PR\left(\%\right)=\left(1-\frac{C_{e}}{C_{i}}\right)\cdot 100\%,$$

where $C_{i}$ and $C_{e}$ are the initial and equilibrium metal concentrations, respectively, mg/L. Then, the optimization process was carried out as described further in the next subsection. In this way, the best values of the pH and adsorbent mass $m_{a}$ were determined for each initial concentration as well as overall. For all three metals, the procedure was carried out in the same way.

In the next step, metal sorption kinetics studies were conducted using the determined pH = 5 and $m_{a}$ equal to 5 g/L with a static method (batch). Aqueous solutions of metals in the amount of 50 mg/L were added to the prepared solutions in three separate flasks. Then, the samples were shaken for a period of 5 to 120 min. After the specified time, samples of the solutions were taken for metal concentration analysis. The sorption capacity of the adsorbent towards metals, q (mg/g), was determined as follows:

(4) $$q=\frac{C_{i}-C_{e}}{m_{a}}V,$$

where $C_{i}$ and $C_{e}$ are the initial and equilibrium metal concentrations, respectively, mg/L; V is the volume of the tested solution, L; and $m_{a}$ is the mass of the adsorbent, g.

The kinetics were modelled using several theoretical models as follows: the pseudo-first-order (PFO) equation, the pseudo-second-order (PSO) equation, the intraparticle diffusion (IPD) equation, and the IPD model with one term (IPD1) according to the equations described in [43]. The final equilibrium concentrations of metals were used to determine the adsorption isotherms. The following isotherms were considered: Freundlich, Langmuir, Langmuir–Freundlich, Elovich, Temkin, and Toth (equations as in [9]). The parameters of the kinetics models and the adsorption isotherms were determined using the nonlinear fitting and least squares method. To estimate the fit quality, the determination coefficient $R^{2}$ was used as well as the standard error of regression as follows:

(5) $$SE=\sqrt{\frac{1}{n-p-1}\sum_{i=1}^{n}{\left(\hat{y}\left(x_{i}\right)-y_{i}\right)}^{2}},$$

where n is the number of data points $\left(x_{i},y_{i}\right)$, x is the independent variable, y is the dependent variable, $\hat{y}$ is the model equation, and p is the number of parameters of the model [43].

To determine the effect of temperature, thermodynamic studies based on standard equations (e.g., [43]) were conducted at 20, 30, 40, and 50 °C and initial heavy metal concentrations ranging from 10 to 250 mg/L. Experiments were also carried out at pH = 5 and adsorbent mass $m_{a}$ = 5 g/L to maintain the optimal sorption conditions.

For all experiments, the concentrations of metals in solutions after sorption were quantitatively determined using an inductively coupled plasma–atomic emission spectrometer (ICP-AES). Three series of measurements were performed for each sample, and the average value was taken.

#### 2.2.5. Optimization of Adsorption Parameters

The Central Composite Circumscribed Design (CCD) method with $\alpha =2^{3/4}\approx 1.68$ was used to select the optimal adsorption parameters with three independent variables: the initial solution pH, initial adsorbate concentration $C_{i}$, and adsorbent mass $m_{a}$. These variables were normalized and coded as $x_{1}$, $x_{2}$, and $x_{3}$, respectively, and then, a 20-point CCD scheme was applied. For each of the 20 sets of independent variables, measurements of the final concentration of each adsorbate were performed, and its percentage removal was calculated. To assess the effect of individual independent variables on the removal efficiency, a second-degree polynomial with respect to variables $x_{1}$, $x_{2}$, and $x_{3}$ was used to describe the modelled percentage removal (MPR) as follows:

(6) $$MPR\left(x_{1},x_{2},x_{3}\right)=a_{0}+a_{1}x_{1}+a_{2}x_{2}+a_{3}x_{3}+a_{4}x_{1}^{2}+a_{5}x_{2}^{2}+a_{6}x_{3}^{2}+a_{7}x_{1}x_{2}+a_{8}x_{2}x_{3}+a_{9}x_{3}x_{1},$$

where $a_{0},\;\;a_{1},\;\;\ldots ,{\;a}_{9}$ are coefficients that were further determined using the least squares method. In the calculations, all combinations of the base functions $1,x_{1},\;\ldots ,{\;x}_{3}x_{1}$ were tested, and the best fit was assumed to be the one that gave the highest value of the adjusted $R^{2}$ value, calculated as $AR^{2}=1-\left(n-1\right)/\left(n-m\right)\left(1-R^{2}\right)$, where n is the number of measurement points (20), m is the number of base functions taken into account, and $R^{2}$ is the coefficient of determination. Solutions that gave coefficients $a_{i}$ burdened with too high p-values were also rejected. In this way, the base functions that had a negligible effect on the quality of the fit were eliminated from further consideration (i.e., some of the coefficients $a_{i}$ were assumed to be equal to zero as a result).

In the next step, an attempt was made to determine the optimal values of independent variables that would give the highest possible percentage removal. For this purpose, the maximum of the MPR function was sought in the considered range of independent variables:

(7) $$MPR\left(x_{1},x_{2},x_{3}\right)=max\Rightarrow x_{1}^{o},x_{2}^{o},x_{3}^{o}\Rightarrow {pH}^{o},C_{i}^{o},m_{a}^{o},$$

where the superscript “o” stands for “optimal”.

Unlike the pH and adsorbate mass, the initial concentration is a variable whose value in natural conditions is an existing quantity and cannot be easily changed; therefore, attempts were also made to select the optimal adsorption conditions (i.e., pH and adsorbent mass $m_{a}$) for a given initial adsorbate concentration $C_{i}$. For this purpose, the geometric locus of the MPR function was determined, giving the highest possible value for a given value $x_{2}$, i.e., the maximum of the $MPR\left(x_{1},x_{2},x_{3}\right)$ was determined, treating $x_{2}$ as a parameter:

(8) $$MPR\left(x_{1},x_{2}=const,x_{3}\right)=max\Rightarrow x_{1}^{bC}\left(x_{2}\right),x_{3}^{bC}\left(x_{2}\right)\Rightarrow {pH}^{bC}\left(C_{i}\right),m_{a}^{\mathrm{bC}}\left(C_{i}\right),$$

where the superscript “bC” means “best for concentration $C_{i}$”. The ${pH}^{\mathrm{bC}}$ and $m_{a}^{\mathrm{bC}}$ values determined in this way depend on the initial concentration. However, in technical conditions, this is inconvenient because it would require tracing $C_{i}$ and adjusting the pH and $m_{a}$ accordingly; therefore, the average percentage removal (APR) was calculated by averaging over the initial concentration in the considered range as follows:

(9) $$APR\left(x_{1},x_{3}\right)=\frac{1}{2\alpha }\int_{-\alpha }^{\alpha }MPR\left(x_{1},x_{2},x_{3}\right)dx_{2},$$

and then the optimal pH and $m_{a}$ values were determined:

(10) $$APR\left(x_{1},x_{3}\right)=max\Rightarrow x_{1}^{\mathrm{bA}},x_{3}^{\mathrm{bA}}\Rightarrow {pH}^{\mathrm{bA}},m_{a}^{\mathrm{bA}},$$

where the superscript “bA” means “best on average”. The values of ${pH}^{\mathrm{bA}}$ and $m_{a}^{\mathrm{bA}}$ determined in this way can be considered optimal for many adsorption processes with various initial adsorbate concentrations within the range considered in this work.

## 3. Results and Discussion

### 3.1. Physicochemical Properties of the Adsorbent

The physicochemical properties of TWAC obtained in the pyrolysis process are presented in Table 1 and Table 2. After adding the TWAC into distilled water, the solution pH increased to 9.05, indicating the absorption of protons. The alkaline nature of the solution is related to the pyrolysis process, which leads to the formation of carbonates and aromatic structures with condensed rings with C–O bonds or the reduction of carboxyl groups [44,45,46]. The presence of negatively charged organic groups, such as COO (carboxylate), OH (hydroxyl), and carbonates bound to the surface of activated carbon, also affects the alkalinity of the coal. The obtained ash content in TWAC is within the range obtained for biochars from wood biomass (approx. 43.2–50.8%). According to [47], this is related to the different lignin and cellulose content in the biomass and the degradation of acidic functional groups during high-temperature pyrolysis, which results in higher ash content and a high pH. Compared to commercial activated carbons, the ash content is relatively high [48]. Jin Zhang et al. (2019) carried out the fast pyrolysis of tobacco stems at 450 °C and obtained a similar ash content (25.5%) [18]. The research results (Table 1) indicate that the volatile substances (21.5%) were not completely evaporated, which may be the reason for the clogging of some pores in the TWAC. This is also indicated by the determined iodine value of the TWAC, which is much lower than that suggested by the determined specific surface area of 875.38 m^2^/g.

Table 2 presents the results of the elemental analysis of C, H, N, S, and O of the tested activated biochar. The obtained content of elemental components is comparable to that for biochar obtained in the pyrolysis process from other types of biomass, e.g., from sunflower husks or pine or spruce bark [49,50]. The content of N and S in TWAC is very low, 1.3% and 0.06%, respectively, which indicates that it should not affect the formation of a corrosive environment in the pyrolysis reactor chamber. Knowledge of the H/C and O/C ratios allows us to determine the diversity of the chemical structure of the tested biochar. The H/C ratio describes the aromaticity of the biochar and its stability, while the O/C ratio allows for a comparison of the biochar richness in oxygen functional groups. Higher H/C and O/C values represent a greater amount of oxygen functional groups [51]. In the case of the tested carbon, low H/C and O/C ratio values were obtained, 0.05 and 0.42, respectively, which indicates the loss of functional groups containing H and O (hydroxyl, carboxyl, and others) due to the dehydration and decarboxylation reactions in the pyrolysis process and a higher degree of carbonization of the tested biochar [52]. Moreover, it is believed that the decrease in the H/C ratio is related to dehydration and the increased aromatization of the organic material surface [47,53,54]. The low H/C ratio suggests that the biochar is strongly thermally modified and has large amounts of unsaturated structures [55].

### 3.2. Structural Characteristics

**Figure 2:**
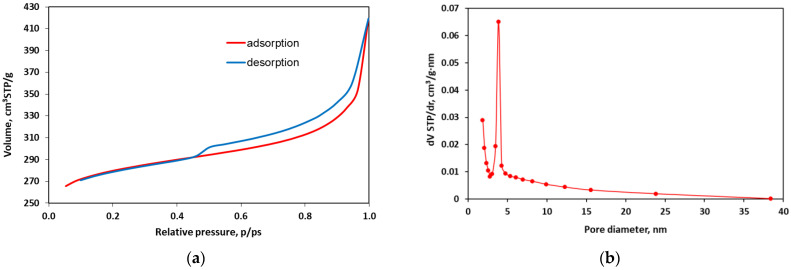
N_2_ adsorption/desorption isotherms (**a**) and pore volume distribution vs. their diameter in the tested activated carbon sample (**b**).

The nitrogen adsorption/desorption isotherms recorded for the tested TWAC sample and the pore volume distribution versus their diameter are shown in Figure 2. According to the IUPAC classification, the isotherm can be classified as type IV with a hysteresis loop of type H3 occurring at higher equilibrium pressures $p/p_{s}=0.5$. However, the entire adsorption branch of the loop of this isotherm seems to have the same shape as the type II isotherm, especially in the range of low and medium $p/p_{s}$ concentrations [56]. Therefore, according to [57], the determined isotherm can rather be classified as pseudo-type II, associated with delayed capillary condensation. This isotherm is characteristic of porous adsorbents with strong adsorbate–adsorbent interactions and corresponds to single- and multilayer physical adsorption. Such isotherms are characteristic of micro- and mesoporous structures with a wide range of pore sizes [58]. The course of adsorption/desorption isotherms also allows for the assessment of the pore shape, since the nature of the hysteresis loop depends on the type of pores present in the adsorbent. According to de Boer [59], the obtained hysteresis loop can be attributed to pores formed between two planes with different mutual inclinations.

The total pore volume of TWAC calculated from water vapor adsorption data is 0.649 cm^3^/g. The large BET surface area (875.38 m^2^/g) and total pore volume indicate the suitability of TWAC in environmental engineering for the removal of heavy metal ions from water and wastewater. This surface area is smaller than that reported for activated carbons but larger than that reported for biochars prepared based on the same raw material. For example, the BET surface area for activated carbon was reported to be 1800 m^2^/g in [60] and 121 m^2^/g in [58], and for biochars, it was 234 m^2^/g in [61] and 310 m^2^/g [62]. Figure 1b shows the pore size distribution of the tested sample determined by the BJH method, which varied from 1 to 10 nm. The graph shows that the dominant pores in the TWAC adsorbent are mesopores with a diameter of about 4 nm. The sizes of hydrated heavy metal ions are 0.430 nm, 0.426 nm, and 0.401 nm for Zn^2+^, Cd^2+^, and Pb^2+^, respectively, which indicates the possibility of multilayer adsorption in both the mesopores and micropores of the adsorbent [63,64].

### 3.3. Point of Zero Charge pH

**Figure 3:**
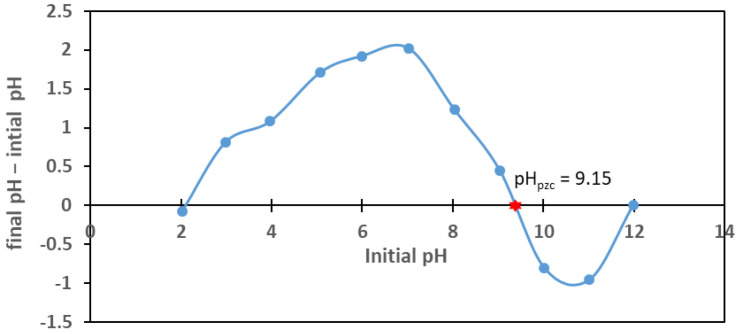
Point of zero charge (pH_pzc_) for the TWAC under study.

The charge of the adsorbent surface plays an important role in the ion sorption process at the solid/solution interface. The adsorption of various substances is highly dependent on the solution’s pH value. A small change in the solution’s pH can lead to a large increase or decrease in the electrostatic interaction between ions, substances, and ionizable sites on the adsorbent surface [65]. When the pH of the solution in contact with the adsorbent is below the point of zero charge (pH_pzc_), the adsorbent surface is positively charged, and conversely, when the pH increases above the pH_pzc_, the surface is negatively charged. Figure 3 shows the results of the pH_pzc_ for the tested biochar sample. The pH_pzc_ value of biochar is 9.15. The high observed pH_pzc_ value is due to the high activation temperature (800 °C) in the pyrolysis process, which results in the formation of carbonates in the TWAC structure [62]. Figure 3 shows that the surface of the adsorbing material is positively charged below the pH value of 9.15; hence, for effective cation sorption, the solution pH should be above that value. However, this is only one of the factors affecting the sorption. In the considered case, such a large pH value would result in precipitation rather than sorption. Therefore, in further studies, the solution pH was lowered to avoid possible precipitation.

The pH_pzc_ values directly depend on the type of activation used, as well as on the chemical properties of the activation solution [66]. The literature shows that biochars and activated carbons obtained by the pyrolysis of biomass exhibit a large variability in the pH_pzc_. For example, in studies on the adsorbent from tobacco stems obtained by activation with ZnCl_2_ and CO_2_ at a temperature of 750 °C, pH_pzc_ = 9.05 was obtained [62], whereas for activated carbon prepared from starch chemically activated in KOH at a temperature of 750 °C, the pH_pzc_ was 5 [67], and for biochar obtained from apple and cherry sawdust at a temperature of 800 °C after chemical activation with KOH, it was 6.6 [68].

### 3.4. FTIR Analysis

**Figure 4:**
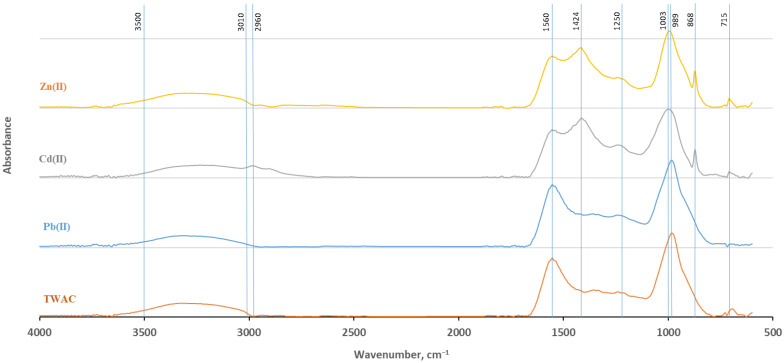
FTIR spectra of the TWAC before and after sorption of Pb(II), Zn(II), and Cd(II).

The identification of surface functional groups present in a material is important for understanding its chemical and sorption properties and adsorption mechanisms [69,70]. The Fourier-transform infrared (FTIR) analysis results indicate qualitative differences in the biochar surface functional groups, which depend on the raw material used and pyrolysis conditions [71]. Figure 4 shows the FTIR spectra of TWAC before and after the adsorption of Pb(II), Cd(II), and Zn(II).

The broad absorption band in the range of 3500–3010 cm^−1^ indicates the presence of hydroxyl groups (–OH) originating from cellulose, hemicellulose, lignin, and other organic compounds and amine groups (–NH) originating from the tobacco stem biomass [58]. The intensity of the band assigned to stretching (–OH) decreased slightly in the case of Pb(II), and after Cd(II) sorption, the peak showed a slight shift, indicating the breakage of O–H bonds (Figure 4). In the case of Pb(II), no other clear changes were observed in the FTIR spectrum after adsorption, suggesting that the adsorption process may take place via physical or electrostatic interactions that do not lead to significant changes in the functional groups of the adsorbent, and these mechanisms are more difficult to capture in the FTIR spectrum [72]. The stretching vibration bands of the –CH groups, in particular the methyl substituents of aromatic rings (–CH_3_) and the methylene bonds connecting aromatic rings (–CH_2_), are present in the spectra of TWAC samples after Zn(II) and Cd(II) adsorption near a wavenumber of 2960 cm^−1^, which suggests that they influence the sorption of these metals [73,74]. However, they are not visible in the spectrum of TWAC after Pb(II) sorption. The band present in the 1560 cm^−1^ region can be attributed to the stretching vibrations of both the C=C groups in aromatic structures and C=O in carboxylates, ketones, and quinones [75,76], with a significantly decreased peak corresponding to C=C and C=O stretching upon Zn(II) and Cd(II) adsorption. The appearance of a new peak around 1424 cm^−1^ (related to aliphatic vibrations such as methyl (–CH_3_) or methylene (–CH_2_) groups and (–COO^−^) groups) indicates the participation of these groups in the removal of Cd(II) and Zn(II) and the formation of complexes with these groups [51,73]. The transformation of –COOH groups into anionic form (–COO^−^) as a result of biomass pyrolysis may lead to the appearance of a peak in the range of 1420–1430 cm^−1^ because carboxyl groups can cause asymmetric vibrations in this region of the FTIR spectrum. A small peak around 1250 cm^−1^ is most often associated with the vibrations of C–O groups in phenolic groups (–OH), as well as ester groups (–C–O–C–) and aliphatic (–CH_2_, –CH_3_) or carbonyl (C=O) groups. The high peak at 1003–989 cm^−1^ may be caused by the stretching vibrations of the C–O bond of alcohol groups and carboxylic acids; its intensity decreased after the adsorption of Zn(II) and Cd(II) [77,78]. On the other hand, the position of the peak at 869 cm^−1^ may be the result of the chelation of Zn(II) and Cd(II) with oxygen groups on the adsorbent surface. This peak is responsible for the out-of-plane vibrations of C–H groups in aromatic, carboxyl (–COOH) in anionic form (–COO^−^), and epoxy (–C–O–C–) groups. The obtained FTIR spectra are consistent with the changes in the elemental composition, which indicates that an increase in the pyrolysis temperature results in the formation of activated carbon with increased aromaticity and decreased acidity and polarity (Table 2) [79].

### 3.5. Optimization of Sorption Conditions

In order to determine the optimal values of the selected parameters in the adsorption process, the CCD method was used. The values of the original independent variables ${pH}_{i}$, $C_{i}$, and $m_{a}$, their normalized equivalents, and the determined percentage removal (PR) are given in Table 3. The removal efficiency of Pb(II) is high and ranges from 82% to 99.5%. However, for the two remaining metal ions, the adsorption efficiency is lower and ranges from about 29% to 99% for Zn(II) and from 40% to 99.5% for Cd(II).

**Figure 5:**
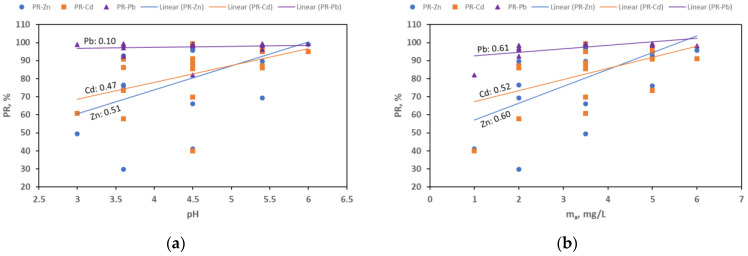
Correlations between the observed values of percentage removal (PR) and the values of the initial pH (**a**) and adsorbent mass $m_{a}$ (**b**).

Figure 5 shows correlation graphs between the obtained values of percentage removal and the values of the initial pH and the mass of the adsorbent. In all cases, a positive correlation was observed, which indicates that approximately higher values of ${pH}_{i}$ and higher values of $m_{a}$ give a higher removal efficiency.

**Figure 6:**
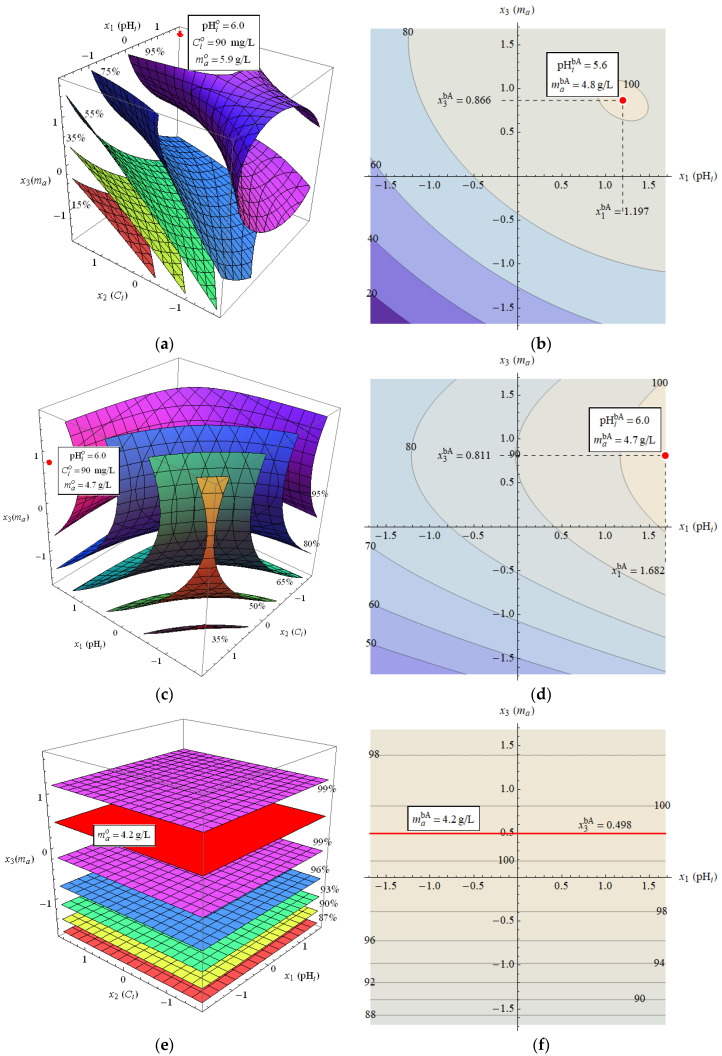
Plots of MPR and APR functions: (**a**) MPR for Zn(II), (**b**) APR for Zn(II), (**c**) MPR for Cd(II), (**d**) APR for Cd(II), (**e**) MPR for Pb(II), and (**f**) APR for Pb(II); the maxima of the functions in the considered range of variables are marked in red; the values of the variables defining the maxima are given in framed white rectangles.

To obtain the optimal ${pH}_{i}$ and $m_{a}$ values for each of the adsorbates studied, the MPR function was used, as described in Section 2.2.5. The determined forms of the MPR function, $AR^{2}$ fit indicator, and APR function for the individual adsorbates are given in Table 4 and illustrated in Figure 6. The most complex MPR dependence was obtained for Zn(II)—it is characterized by a high fit quality and contains both linear and quadratic terms, as well as mixed terms. For Cd(II), the MPR function is slightly less complex but is characterized by a poorer quality of fit. In the case of Pb(II), the MPR function is extremely simple and seems to depend only on the adsorbent mass, but its quality of fit is quite poor. It should be emphasized that the determined functions are empirical in nature and do not result directly from the adsorption mechanism. Nevertheless, they are helpful in selecting the optimal adsorption conditions.

Based on the form of the MPR function, the position of its maximum was determined, located in the studied range of variables, corresponding to the optimal values of the adsorption parameters, which are shown in Table 5.

In the case of Zn(II), the percentage removal varies in the range from about 10% to 100% and is approximately greater the higher the ${pH}_{i}$ values and the mass of the adsorbent. The best adsorption conditions resulting from the MPR function are ${pH}_{i}^{o}=6.0$, $C_{i}^{o}=90\;\mathrm{mg}/L$, and $m_{a}^{o}=5.9\;g/L$, but it should be noted that MPR values close to 100% occur for a mass of the adsorbent above 4 g/L and ${pH}_{i}$ above 4.5. The averaged optimal ${pH}_{i}$ and $m_{a}$ values over the concentrations that maximize the APR function are 5.6 and 4.8 g/L, respectively.

The percentage removal of Cd(II) is characterized by high variability (20–100%) and is clearly positively correlated with the adsorbent mass and pH value. Unlike Zn(II), the optimal adsorbent mass is independent of $C_{i}$ and equals about 4.7 g/L. As for the initial pH, for low cadmium concentration values, the ${pH}_{i}$ value seems to be of little importance, but for higher concentrations, better results are obtained for ${pH}_{i}$ close to 6.0. Values of the percentage of removal close to 100% are obtained for ${pH}_{i}$ above 5.0 and $m_{a}$ above 3.5 g/L. The averaged optimal values for ${pH}_{i}$ and $m_{a}$ are 6.0 and 4.7 g/L, respectively.

In the case of Pb(II), the percentage removal varies from about 85 to 100%. The adsorbent mass has a significant effect on the removal percentage—its optimal value is about 4.2 g/L, but MPR values close to 100% are also obtained for masses above about 3.5 g/L. In turn, the influence of the $C_{i}$ and ${pH}_{i}$ values is relatively small in the considered range of their variability (hence, the MPR function lacks variables $x_{1}$ and $x_{2}$, and there is only $x_{3}$ corresponding to the adsorbent mass).

Comparing the optimal values of ${pH}_{i}$ and $m_{a}$ for all three adsorbates, it can be seen that they are similar, which is especially visible in the case of the values of ${pH}_{i}^{\mathrm{bA}}$ and $m_{a}^{\mathrm{bA}}$. As indicated by the above analysis of the variability in the MPR and APR functions, small deviations from these values do not significantly affect the adsorption efficiency. Therefore, it seems reasonable to choose one common value for each of ${pH}_{i}$ and $m_{a}$ that would give high values of the removal percentage for all three adsorbates. Based on the above analysis, such values can be assumed as ${pH}_{i}=5–6$ and $m_{a}=4–5\;g/L$. In order to avoid possible ion precipitation in further studies, ${pH}_{i}=5.0$ was assumed. As for the adsorbent mass, $m_{a}=5\;g/L$ was chosen.

### 3.6. Sorption Kinetics—The Effect of Contact Time

**Figure 7:**
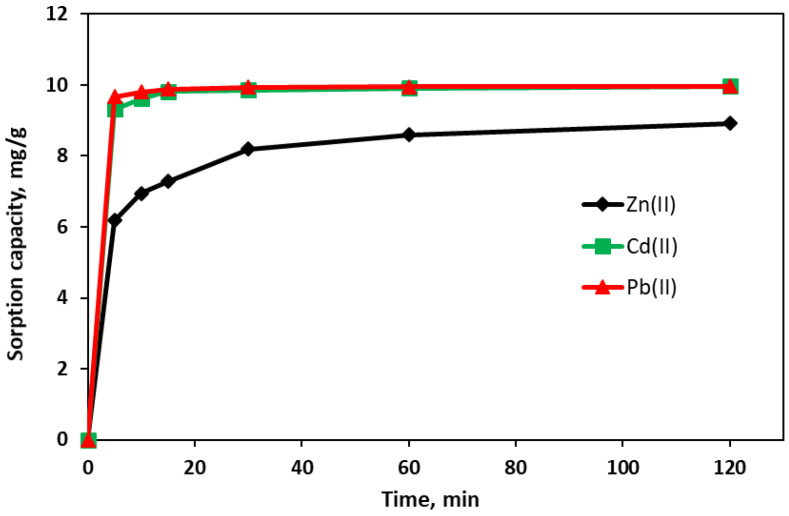
Adsorption kinetics of Zn(II), Cd(II), and Pb(II) on TWAC at the initial metal concentration of 50 mg/L.

The results of the adsorption kinetics of Zn(II), Cd(II), and Pb(II) on the TWAC are presented in Figure 7 and Table 6. The studies showed that the amount of heavy metals removed from the solution increased with an increase in the shaking time of the samples. Initially, the sorption process was very fast, with the adsorption rate being higher for Pb(II) and Cd(II) (Figure 7), for which 90% of the equilibrium value was achieved after about 2 min, while for Zn(II), about 60% of the equilibrium adsorption value was achieved after around 5 min. After this time of contact with the adsorbent, Pb(II) and Cd(II) showed similar values of sorption capacity, 9.67 mg/g and 9.33 mg/g, respectively. They are significantly higher than that for Zn(II) of 6.18 mg/g.

The kinetic curves describe the course of the adsorption process from the initial state to the equilibrium state in terms of quality and quantity and indicate the direction of adsorption. The shape of the kinetic curves for Pb(II) and Cd(II) sorption in the initial phase of the adsorption process indicates a strong interaction between the active sites located on the adsorbent surface and Pb(II) and Cd(II) ions. In the case of Zn(II) sorption, the curve shows that its ions gradually occupied the adsorbent surface until the adsorption equilibrium state was reached after about 120 min. Over time, the active sites on the TWAC surface were completely saturated with the adsorbate, and the experimental curves reached the saturation state of the TWAC exchange complex (Figure 7).

The parameters of the considered adsorption kinetics models are presented in Table 6. In the PFO and PSO models, a better agreement in fitting the results was obtained than in the case of the IPD and IPD1 models. For the tested metals, the obtained results of the adsorption rate constants in the PFO, PSO, and IPD1 models are similar. No significant differences were observed between the adsorption rate constants, especially for Cd(II) and Pb(II); slightly lower values were noted for Zn(II) (Table 6). However, the PSO model obtained a lower regression standard error for Zn(II) (SE = 0.32 mg/g), Cd(II) (SE = 0.04 mg/g), and Pb(II) (SE = 0.01 mg/g) compared to PFO, which suggests that the adsorption rate of metals on the activated carbon was controlled by the chemical adsorption mechanism due to surface adsorption interactions between heavy metal ions and functional groups [80,81]. According to [82], the PSO model describes the external diffusion in the boundary layer, surface adsorption, and intramolecular diffusion processes. The half-saturation times predicted by the models are very small and range from about 15 s to about 2 min for Pb(II) and Cd(II), whereas they range from about 3 to 8 min for Zn(II). This means that Pb(II) and Cd(II) have a higher mobility in solution due to smaller hydration complexes, so they pass through the boundary layer faster, whereas Zn(II) interacts less strongly with the adsorbent, which slows down its adsorption.

In the IPD model, the obtained C values for the intersection point are in each case greater than zero (Table 6), which suggests that intraparticle diffusion is involved in the adsorption process; however, this is not the rate-controlling step of the adsorption process. The constant related to the thickness of the boundary layer increases in the order Zn(II) < Cd(II) < Pb(II), which indicates that Pb(II) and Cd(II) are more strongly adsorbed on the adsorbent surface than Zn(II). In the case of Pb(II) and Cd(II), a higher C value suggests a greater influence of the boundary layer on the adsorption process, which may result from a higher chemical affinity for the adsorbent and weaker hydration. The calculated intraparticle diffusion rate constants in the IPD model are similar for the individual metals—about 0.6 mg/g min^−0.5^. The adsorption mechanism of Zn(II), Cd(II), and Pb(II) depends on both the boundary layer (IPD1) and the internal diffusion (IPD). According to [83], the fast adsorption of metal ions in the initial stage is controlled by the boundary layer and intramolecular diffusion, while in the later stage, the slow adsorption can be controlled by intramolecular diffusion.

### 3.7. Adsorption Isotherms

**Figure 8:**
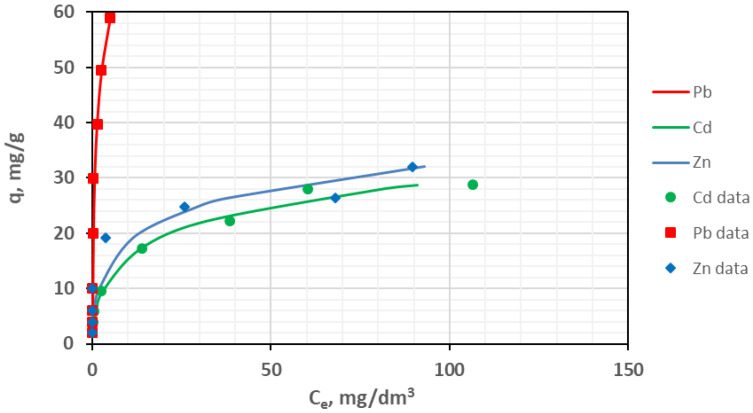
Elovich adsorption isotherms for Zn(II), Cd(II), and Pb(II) on the tested material.

The parameters of several adsorption isotherms for the considered metal ions on the tested activated carbon are presented in Table 7. Isotherms corresponding to the nonlinear Elovich model are shown in Figure 8.

Comparing the adsorption of heavy metals on TWAC, it was found that the amount of metals bound by the adsorbent decreased in the order Pb(II) > Zn(II) > Cd(II) (Figure 8). A similar trend, regardless of the type of biochar used, was observed in [84], where the sorption of Pb(II), Zn(II), and Cd(II) on biochars derived from rapeseed straw, wheat straw, miscanthus, and soft coal was analyzed. The process of element sorption was described by the Hill model isotherms in the monometallic system for initial concentrations from 0.05 to 50 mg/L for Cd(II) and Zn(II) and from 0.5 to 85 mg/L for Pb(II).

At the maximum initial concentration, the adsorbed amounts were 60 mg/g for Pb(II), 32 mg/g for Zn(II), and 28.7 mg/g for Cd(II). The obtained adsorption capacity values are slightly lower compared to the results obtained in [85], in which sorption was carried out on biochar also obtained from tobacco stems in a pyrolysis process at 700 °C for 6 h, and the Pb(II) and Cd(II) contents were approximately 58 mg/g and 70 mg/g, respectively. On the other hand, a similar sorption capacity (approx. 60 mg/g) was obtained for Pb(II) in [62] for biochar obtained by chemical activation using ZnCl_2_ + CO_2_ and pyrolysis at 750 °C.

The parameter values of the isotherms indicate that the adsorption mechanism differs for individual metals. The coefficients of determination R^2^ for Zn(II) range from 0.748 to 0.982, with the best fit obtained for the Elovich isotherm and slightly worse fits for the Freundlich and Temkin models. This suggests that the sorption mechanism is mixed. In addition to chemisorption, which is described by the Elovich model, physical interactions of Zn(II), such as electrostatic interactions and multilayer adsorption, are also possible. Furthermore, Zn(II) can bind by complexation with the functional groups of activated carbon and interact electrostatically.

The Elovich model also best describes Pb(II) sorption, which is confirmed by the highest coefficient of determination (R^2^ = 0.974). Analysis of the sorption isotherms indicates that Pb(II) is mainly adsorbed via chemisorption, as indicated by the good fit of the Elovich model and high adsorption capacity in the Langmuir model ($Q_{L}$ = 64.23 mg/g) and Langmuir–Freundlich models ($Q_{LF}$ = 107.95 mg/g). The probable mechanism of Pb(II) sorption includes complexation with the functional groups of the adsorbent, ion exchange, and electrostatic interactions. Pb(II) shows the highest adsorption capacity, which indicates strong chemical interactions, and the high $Q_{E}$ value suggests that the sorption is strongly dependent on the activation energy.

In turn, the Toth and Freundlich isotherms best describe Cd(II) sorption—the determination coefficients are 0.992 and 0.987, respectively. The Toth model describes the adsorption on heterogeneous surfaces, where the process is more complex and involves different interaction mechanisms, which suggests a different affinity of Cd(II) to the adsorbent. On the other hand, the Freundlich model indicates the multilayer adsorption of Cd(II), which suggests the presence of both chemisorption and physisorption on the heterogeneous adsorbent surface [86].

### 3.8. Sorption Thermodynamics

**Figure 9:**
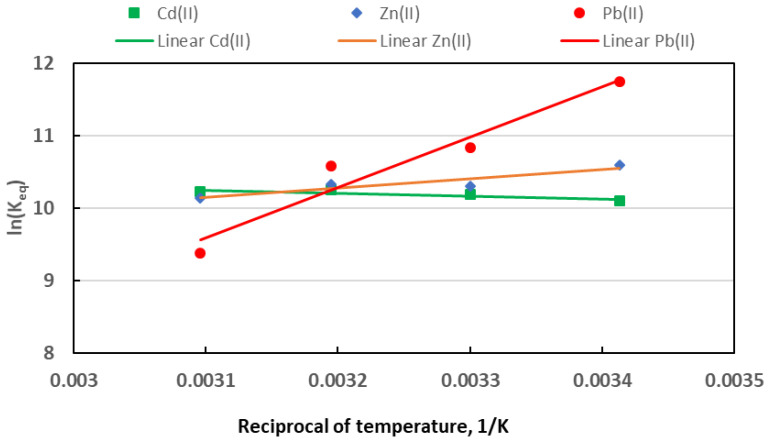
Results of thermodynamic studies of Zn(II), Cd(II), and Pb(II) sorption on TWAC.

Sorption thermodynamics studies allow the prediction of sorption efficiency in various conditions, which is crucial in designing water and sewage treatment technologies. Temperature affects the adsorption efficiency of metal ions in the adsorption process. Temperature change causes changes in basic thermodynamic parameters, such as free energy change (ΔG), enthalpy change (ΔH), and entropy change (ΔS) [87]. The results of the thermodynamic analysis of metal adsorption are presented in Table 8 and Figure 9.

In each case, the change in the Gibbs free energy (ΔG) is negative, which indicates that adsorption occurs spontaneously. With an increase in temperature, the value of the $K_{eq}$ constant, determined using the Langmuir equation, clearly decreased for Pb(II) and slightly decreased for Zn(II), whereas it increased insignificantly for Cd(II) (Figure 9). This shows that temperature did not significantly affect Cd(II) and Zn(II) sorption. Negative values of the enthalpy change in the Pb(II) and Zn(II) sorption indicate the exothermic nature of the process. Similar values of the enthalpy change were obtained for the Pb(II) adsorption process on the adsorbent prepared from coconut shells modified with polysiloxane [88]. In turn, in the case of Cd(II), the enthalpy change is positive, which, combined with the positive value of the entropy change ΔS, means that Cd(II) sorption on TWAC is an endothermic process, occurring better at higher temperatures. Negative values of ΔG were also obtained for the adsorption of Pb(II) ions on activated carbon obtained from tamarind wood in [89]. In the case of Pb(II) adsorption, the ΔH value is below −40 kJ/mol, which confirms the chemical nature of its sorption process, whereas in the case of Zn(II) and Cd(II), the ΔH value is above −20 kJ/mol, which indicates the physical nature of the adsorption.

### 3.9. Comparative Studies

The properties of the activated carbon used in this study were compared with those reported in the literature (Table 9). The maximum sorption capacity for Zn(II), Cd(II), and Pb(II) on the tested adsorbent is comparable to that for other biosorbents derived from agricultural waste and wood materials. This indicates the potential of this waste material as an effective sorbent for the removal of heavy metals in water and soil purification processes. Although some biomaterials such as rapeseed straw achieve better sorption results, especially for Cd(II), tobacco waste still remains a material worth further research, especially in the context of Pb(II) and Zn(II) removal. In addition, its availability and ease of processing into activated carbon make it an interesting alternative in the development of innovative water treatment technologies.

## 4. Conclusions

Based on the research conducted and results obtained, the following conclusions regarding the adsorption of Zn(II), Cd(II), and Pb(II) on activated biochar obtained from waste tobacco stems can be formulated:

- The research conducted on the selection of optimal parameters for the adsorption of Zn(II), Cd(II), and Pb(II) indicate that the mass of the adsorbent and the initial pH should be 4–5 g/L and pH = 5–6, respectively. To avoid possible ion precipitation, the suggested pH value is 5.0.
- The amount of adsorbed compounds depends on the type of metal and satisfies the approximate relationship Pb(II) > Zn(II) > Cd(II).
- The results of the adsorption kinetics indicate that the adsorption process of Pb(II) and Cd(II) is very fast—after a few minutes, the adsorbent is practically saturated. In the case of Zn(II), the adsorption process is much slower and takes about two hours. The kinetics seem best described by a pseudo-second-order model, which indicates that in addition to physical sorption, adsorption includes chemical interactions leading to the binding of metal ions on the adsorbent surface via mechanisms such as ion exchange or complexation.
- Pb(II) adsorption occurs most strongly, mainly through chemisorption, while Zn(II) and Cd(II) sorption shows a more complex mechanism including both chemisorption and physical adsorption.
- The adsorption occurs spontaneously for all the tested metals; the process is exothermic for Zn(II) and Pb(II), whereas it is endothermic for Cd(II).

Further studies will be focused on assessing the effectiveness of the obtained adsorbent in the sorption of heavy metals from real industrial wastewater. It is also worth investigating the effect of surface modification on the selectivity of adsorption and analyzing the profitability of the entire process on a semi-technical or industrial scale. The use of tobacco waste as a low-cost precursor is consistent with the assumptions of the circular economy and creates prospects for the development of sustainable adsorbents for applications in water purification and environmental remediation.

## Figures and Tables

**Table 1:** Physicochemical characteristics of the activated carbon obtained from tobacco waste.

**Quantity**	pH	$\mathrm{Moisture}\;\mathrm{Content}\;(H_{a})$	$\mathrm{Ash}\;\mathrm{Content}\;(A_{a})$	Volatile Matter (VM)	Density	BET Surface	Iodine Value	Methylene Blue Index
**Unit**		%	%	%	g/cm^3^	m^2^/g	mg/g	cm^3^
**Value**	9.05	1.94	26.8	21.5	0.713	875.38	285.6	20

**Table 2:** Elemental analysis of the activated carbon obtained from tobacco waste.

**Quantity**	C (%)	H (%)	N (%)	S (%)	O (%)	H/C	O/C
**Value**	49	2.5	1.3	0.06	20.34	0.05	0.42

**Table 3:** Input and output data for removal optimization with CCD.

${pH}_{i}$	$x_{1}$	$C_{i},$ mg/L	$x_{2}$	$m_{a},$ g	$x_{3}$	$C_{e},mg/L$			Removal (***PR***), %		
						Zn	Cd	Pb	Zn	Cd	Pb
3.6	−1	26	−1	0.2	−1	6.16	3.59	1.97	76.5	86.3	92.5
5.4	1	26	−1	0.2	−1	2.70	3.37	0.97	89.7	87.1	96.3
3.6	−1	74	1	0.2	−1	51.89	31.2	2.12	29.7	57.8	97.1
5.4	1	74	1	0.2	−1	22.63	10.44	1.0	69.3	85.9	98.6
3.6	−1	26	−1	0.5	1	1.88	2.4	0.39	92.8	90.8	98.5
5.4	1	26	−1	0.5	1	0.55	0.73	0,25	97.9	97.2	99.0
3.6	−1	74	1	0.5	1	17.73	19.54	0.47	76.0	73.5	99.4
5.4	1	74	1	0.5	1	1.45	3.61	0.38	98.0	95.1	99.5
3.0	0	50	0	0.35	0	25.31	19.66	0.52	49.4	60.7	99.0
6.0	0	50	0	0.35	0	0.48	2.45	0.23	99.0	95.1	99.5
4.5	0	10	0	0.35	0	0.32	0.05	0.13	96.8	99.5	98.7
4.5	−1.68	90	0	0.35	0	30.59	27.21	0.62	66.0	69.8	99.3
4.5	1.68	50	0	0.1	0	29.44	30.02	0.99	41.1	40	82
4.5	0	50	−1.68	0.6	0	2.11	4.42	0.88	95.8	91.2	98.2
4.5	0	50	1.68	0.35	0	5.09	5.60	0.39	89.8	88.8	99.2
4.5	0	50	0	0.35	−1.68	6.35	7.24	0.53	87.3	85.5	98.9
4.5	0	50	0	0.35	1.68	5.51	6.31	0.44	89.0	87.4	99.1
4.5	0	50	0	0.35	0	5.95	6.93	0.41	88.1	86.1	99.2
4.5	0	50	0	0.35	0	6.21	5.86	0.49	87.6	88.3	99.0
4.5	0	50	0	0.35	0	5.72	7.11	0.51	88.6	85.8	99.0

**Table 4:** Functions describing the empirical percentage removal of Zn(II), Cd(II), and Pb(II) on the activated carbon under study; $x_{1}$, $x_{2}$, and $x_{3}$ are normalized variables representing ${pH}_{i}$, $C_{i}$, and $m_{a}$.

Adsorbate	Function or Indicator	Value
Zn(II)	MPR	$87.05+11.97x_{1}-9.93x_{2}+14.02x_{3}-3.84x_{1}^{2}-5.87x_{3}^{2}+5.43x_{1}x_{2}-3.22x_{1}x_{3}+6.31x_{2}x_{3}$
	$AR^{2}$	0.942
	APR	$87.05+11.97x_{1}+14.02x_{3}-3.84x_{1}^{2}-5.87x_{3}^{2}-3.22x_{1}x_{3}$
Cd(II)	MPR	$86.47+8.40x_{1}-7.27x_{2}+9.21x_{3}-5.68x_{3}^{2}+5.31x_{1}x_{2}$
	$AR^{2}$	0.765
	APR	$86.47+8.40x_{1}+9.21x_{3}-5.68x_{3}^{2}$
Pb(II)	MPR	$99.57+2.86x_{3}-2.88x_{3}^{2}$
	$AR^{2}$	0.738
	APR	$99.57+2.86x_{3}-2.88x_{3}^{2}$

**Table 5:** Optimal values of adsorption parameters.

Adsorbate	Optimal Conditions for Sorption	Best Parameters for Given Concentration	Best Parameters on Average
Zn(II)	${pH}_{i}^{o}=6.00$ $C_{i}^{o}=90\mathrm{mg}/L$ $m_{a}^{o}=5.9\;g/L$	${pH}_{i}^{\mathrm{bC}}=4.55+0.0204C_{i}$ $m_{a}^{bC}=3.8+0.0242C_{i}$	${pH}_{i}^{\mathrm{bA}}=5.6$ $m_{a}^{bA}=4.8\;g/L$
Cd(II)	${pH}_{i}^{o}=6.00$ $C_{i}^{o}=90\mathrm{mg}/L$ $m_{a}^{o}=4.7\;g/L$	${pH}_{i}^{\mathrm{bC}}=6.0$ $m_{a}^{bC}=4.7\;g/L\;$	${pH}_{i}^{\mathrm{bA}}=6.0$ $m_{a}^{bA}=4.7\;g/L$
Pb(II)	${pH}_{i}^{o}=3.0–6.0$ $C_{i}^{o}=10–90\mathrm{mg}/L$ $m_{a}^{o}=4.2\;g/L$	${pH}_{i}^{\mathrm{bC}}=3.0–6.0$ $m_{a}^{bC}=4.2\;g/L$	${pH}_{i}^{\mathrm{bA}}=3.0–6.0$ $m_{a}^{bA}=4.2\;g/L$

**Table 6:** Results of the analysis of the adsorption kinetics of Zn(II), Cd(II), and Pb(II).

Parameter or Indicator	Unit	Zn(II)	Cd(II)	Pb(II)
Pseudo-first-order (PFO) model: $dq/dt=k_{1}\left(Q_{e}-q\right)$				
$Q_{e}$	mg/g	8.22	9.84	9.91
$k_{1}$	min^−1^	0.24	0.58	0.73
$t_{1/2}$	min	2.87	1.19	0.95
SE	mg/g	0.61	0.11	0.06
Pseudo-second-order (PSO) model: $dq/dt=k_{2}{\left(Q_{e}-q\right)}^{2}$				
$Q_{e}$	mg/g	8.83	9.98	9.97
$k_{2}$	min^−1^	0.045	0.28	0.60
$t_{1/2}$	min	2.49	0.35	0.17
SE	mg/g	0.32	0.02	0.01
Intraparticle diffusion (IPD) model: $q=k_{i}\sqrt{t}+C$				
$k_{i}$	mg/g min^−0.5^	0.67	0.64	0.62
C	mg/g	3.33	5.26	5.42
SE	mg/g	2.03	3.15	3.25
Intraparticle diffusion model with one term (IPD1): $q=\left(1-6/\pi^{2}\exp\left(-Bt\right)\right)$				
$Q_{e}$	mg/g	8.02	9.54	9.62
B	min^−1^	0.17	0.67	2.73
$t_{1/2}$	min	7.19	1.78	0.44
SE	mg/g	1.52	1.70	1.71

**Table 7:** Isotherm constants for the adsorption of Zn(II), Cd(II), and Pb(II) on the tested activated carbon.

Parameter	Unit	Zn(II)	Cd(II)	Pb(II)
$\mathrm{Freundlich}\;\mathrm{isotherm}:\;q=K_{F}C_{e}^{1/n_{F}}$				
*K*_F_	mg/g (L/mg)^1/*n*F^	12.28	7.31	64.41
*n*_F_	–	4.69	3.26	1.58
SE	mg/g	2.98	1.323	10.63
*R* ^2^	–	0.945	0.987	0.906
$\mathrm{Langmuir}\;\mathrm{isotherm}:\;q=Q_{L}\left(K_{L}C_{e}\right)/\left(1+K_{L}C_{e}\right)$				
*K*_L_	L/mg	3.67	0.16	1.51
*Q*_L_	mg/g	26.82	28.72	64.23
SE	mg/g	4.62	2.689	5.69
*R* ^2^	–	0.868	0.947	0.93
$\mathrm{Langmuir}–\mathrm{Freundlich}\;\mathrm{isotherm}:\;q=Q_{L}{\left(K_{L}C_{e}\right)}^{n_{LF}}/\left(1+{\left(K_{L}C_{e}\right)}^{n_{LF}}\right)$				
*K*_LF_	L/mg	0.034	0.034	2.362
*Q*_LF_	mg/g	26.06	21.13	107.95
*n*_LF_	–	34.16	34.16	1.389
*R* ^2^	–	0.748	0.621	0.943
$\mathrm{Elovich}\;\mathrm{isotherm}:\;q/Q_{E}=K_{E}C_{e}\exp\left(-q/Q_{E}\right)$				
*K*_E_	L/mg	2.23	1.228	2.88
*Q*_E_	mg/g	8.11	8.27	68.35
*R* ^2^	–	0.982	0.936	0.974
$\mathrm{Temkin}\;\mathrm{isotherm}:\;q=K_{T}\ln\left(A_{T}C_{e}\right)$				
*K*_T_	mg/g	3.05	3.57	13.69
*A*_T_	L/mg	179.17	15.96	15.54
*R* ^2^	–	0.921	0.927	0.957
$\mathrm{Toth}\;\mathrm{isotherm}:\;q=A_{T}C_{e}/{\left(B_{T}+C_{e}^{m_{T}}\right)}^{1/m_{T}}$				
*A*_T_	mg/g	148.02	165.86	65.11
*B*_T_	mg/g	0.301	0.637	0.666
m_T_	–	0.109	0.156	0.971
*R* ^2^	–	0.949	0.992	0.937

**Table 8:** Results of thermodynamic studies of Zn(II), Cd(II), and Pb(II) adsorption on activated carbon prepared from tobacco waste.

Temperature, *T*, °C	The Gibbs Free Energy Change (ΔG), kJ/mol		
	Cd(II)	Zn(II)	Pb(II)
20	−24.67	−25.70	−28.65
30	−25.63	−26.21	−27.66
40	−26.58	−26.72	−26.66
50	−27.53	−27.23	−25.67
ΔH*,* kJ/mol	3.3 ± 1.3	−10.69 ± 3.32	−57.73 ± 10.05
ΔS*,* kJ/(mol K)	0.095 ± 0.004	−0.051 ± 0.01	−0.099 ± 0.03

**Table 9:** Comparison of sorption results on various biosorbents.

Biomass Raw Material	Activation Method	Adsorbate	Maximum Sorption Capacity, mg/g	Reference
Tobacco stems	Pyrolysis at 800 °C + modification with KOH	Zn Cd Pb	32 28.7 60	This study
Sorghum straw	Slow pyrolysis at 600 °C	Cd Pb	29 125	[90]
Olive branches	Pyrolysis at 450 °C + activation with H_3_PO_4_	Zn Cd Pb	34.97 38.17 41.32	[91]
Walnut shell	Pyrolysis at 400 °C + modification with KMnO_4_	Zn Cd Pb	58.96 44.94 70.37	[92]
Rice straw	Pyrolysis at 450 °C + modification with chitosan and pyromellitic anhydride (PMDA)	Cd Pb	≈30 ≈9	[93]
Oedogonium biomass	Pyrolysis at 600 °C	Zn Cd	13.70 9.11	[94]
Rape straw	Pyrolysis at 600 °C	Cd	81.1	[95]
Hemp fibers	Room temperature + modification with 17.5% NaOH + boiling temperature + modification with 0.7% NaClO_2_	Zn Cd Pb	2.29 3.93 15.32	[96]
Cotton straw (*Gossypium* sp. L.)	Pyrolysis at 500 °C	Cd Zn	≈8.7 ≈6	[97]

## Data Availability

The original contributions presented in this study are included in the article. Further inquiries can be directed to the corresponding author.

## Abbreviations

The following abbreviations are used in this manuscript:

APR	Average percentage removal
CCD	Central Composite Design (Circumscribed)
FTIR	Fourier-transform infrared
IPD	Intraparticle diffusion
IPD1	Intraparticle diffusion with one term
MPR	Modelled percentage removal
PFO	Pseudo-first order
PR	Percentage removal
PSO	Pseudo-second order
SE	Standard error of regression
TWAC	Tobacco waste activated carbon
VM	Volatile matter
